# Neurovascular retinal impairment in early-treated adults with phenylketonuria

**DOI:** 10.3389/fneur.2024.1305984

**Published:** 2024-06-21

**Authors:** Rosa Buonamassa, Giacomo Boscia, Marida Gaudiomonte, Silvana Guerriero, Rita Fischetto, Alfonso Montepara, Maria Oliva Grassi, Maria Grazia Pignataro, Pasquale Puzo, Ermete Giancipoli, Marina D’addario, Giovanni Alessio, Francesco Boscia, Pasquale Viggiano

**Affiliations:** ^1^Department of Translational Biomedicine Neuroscience, University of Bari “Aldo Moro”, Bari, Italy; ^2^Metabolic Diseases and Clinical Genetics Unit, Department of Pediatric Medicine, Giovanni XXIII Children’s Hospital, Bari, Italy; ^3^Department of Ophthalmology, University of Foggia, Foggia, Italy

**Keywords:** early-treated adults with phenylketonuria, phenylketonuria, pRNFL, radial peripapillary capillary plexus, OCTA, retinal imaging

## Abstract

**Purpose:**

To compare radial peripapillary capillary (RPC) vascular plexus parameters and peripapillary retinal nerve fiber layer (pRNFL) thickness between Early-Treated Adults with Phenylketonuria (ETPKU) and controls.

**Methods:**

This observational study was a monocentric, case control study including 36 eyes of 36 participants. Among these, 18 were early-treated PKU (ETPKU) and 18 were controls. A SD-OCTA (XR Avanti AngioVue OCTA; Optovue Inc., Fremont, CA) was employed to assess the OCT and OCTA parameters of all the participants. The main outcome measures were the RPC vessels density (VD) %, and the pRNFL thickness.

**Results:**

The average pRNFL thickness was significantly reduced in ETPKU (110.78 ± 12.48 μm) compared to controls (113.22 ± 13.95 μm), *p* = 0.046. The mean VD% of the small vessels of the RPC plexus was 52.31 ± 2.2 in ETPKU and 50.71 ± 3.2 in controls (*p* = 0.049), while the VD% of all the radial peripapillary capillary plexus (RPCP) was 58.5 ± 2.2 in ETPKU and 55.08 ± 3.4 in controls (*p* < 0.001). By contrast, there were no differences in age, sex, and IOP between the two groups.

**Conclusion:**

Through structural OCT and OCTA, we observed thinning of the nerve fibers accompanied by an increase in perfusion of the RPC plexus. Thus, our conclusions suggest that OCTA may serve as a noninvasive method to identify novel retinal biomarkers in ETPKU.

## Introduction

Phenylalanine hydroxylase (PAH) deficiency, conventionally identified as Phenylketonuria (PKU), is an autosomal recessive inherited metabolic disorder marked by various mutations in the gene (1). PAH serves as a catabolic enzyme responsible for converting L-phenylalanine (L-Phe) into L-tyrosine (L-Tyr), a process reliant on molecular oxygen, iron, and tetrahydrobiopterin (BH4) (2). Such deficiency leads to an impaired degradation of L-Phe and its metabolites, resulting in elevated levels in the blood. The hyperphenylalaninaemia and the increased Phe: Tyr ratio leads to Phe and Tyr to compete to cross the blood–brain barrier and tissues of affected individuals, resulting in an accumulation of Phe in the brain, especially in the White Matter (3). The prevalence of PKU varies worldwide, with an incidence of 1:10,000 newborn in Europe (4). As PKU has autosomal recessive inheritance, consanguineous marriage is an important risk factor; thus, high disease incidence is expectable for countries with a high rate of consanguineous marriages (5).

Untreated PKU can lead to a severe neurological impairment and progressive intellectual disability, accompanied by several additional symptoms such as eczematous rash, epilepsy, autism, seizures, and motor deficits (6). The current classification of PKU distinguishes less severe forms of PAH deficiency, referred to as moderate PKU, mild PKU, mild hyperphenylalaninaemia (HPA) or benign HPA, and more severe manifestations referred to as classic PKU (2). The neuropathology is determined by the increased levels of L-Phe, leading to disruptions in neuronal dendritic outgrowth and synaptic connectivity, as well as impaired cerebral glucose metabolism and neurotransmitters deficiency. The primary marker of disease progression includes white matter lesions (WMLs), which correlate with both metabolic control and the patient’s age (2).

To date, the mainstay of the treatment involves limiting dietary phenylalanine intake, coupled with daily supplementation of Phe-free amino acids. Indeed, the introduction of the neonatal screening allowed early intervention for this condition, with a consequent prevention of most of neuropsychological complications and an improved quality of life (3). By consequence, individuals diagnosed with PKU and subjected to lifelong therapy from birth have significantly reduced the incidence of the more severe impairments associated with untreated PKU. This specific group is termed early-treated PKU (ETPKU) (7).

In such a scenario, ocular findings include photophobia, ocular hypopigmentation, cataract and corneal opacities (8), and various functional visual impairment such as a lower Best-corrected visual acuity (BCVA) and a lower contrast sensitivity (9). The introduction of optical coherence tomography (OCT) has facilitated the identification of retinal neurodegeneration across various systemic and ocular conditions (10). Specifically, earlier researchers have directed their attention towards assessing the thickness of the retinal nerve fiber layer (pRNFL) and the ganglion cell layer (GCC), proposing these measurements as potential biomarkers for neurodegeneration (11).

Furthermore, due to the recent advancements in ophthalmic imaging techniques, notably OCTA, the ability to visualize vascular disorders has expanded to encompass various neurodegenerative conditions, including Parkinson’s disease (12) (PD).

Given the retina’s status as an extension of the central nervous system and shares numerous common characteristics with the central nervous system’s vasculature, the potential identification of retinal structural changes through OCT and vascular alterations using OCTA in ETPKU could potentially provide insights into overarching cerebral modifications that manifest during the initial stages of the disease. Consequently, the primary objective of this study was to explore changes in pRNFL thickness and the radial peripapillary capillary (RPC) plexus in individuals with ETPKU when contrasted with controls.

## Materials and methods

### Study participants

This retrospective observational study was a monocentric, case control study including 36 eyes of 36 participants. Among these, 18 patients had ETPKU and were receiving treatment exclusively through dietary protein restriction, while the remaining 18 participants served as controls. ETPKU patients were recruited from Genetics Department of University of Bari “Aldo Moro” and were addressed for the ophthalmological assessment at the Department of Ophthalmology, University of Bari “Aldo Moro,” Italy, spanning from October 2022 to April 2023. The current investigation was performed in compliance with the tenets of the Declaration of Helsinki for research involving human subjects and approved by the Ethical Committee of this study is retrospective in nature, and in accordance with Italian regulations, ethical approval is not necessary for such studies. Instead, only notification to the ethics committee is required. We reached out to all enrolled individuals and their parents to secure their written informed consent for the retrospective utilization of their clinical information.

All patients underwent a comprehensive ophthalmological examination, including best-corrected visual acuity (BCVA), two consecutive measurements of intraocular pressure (IOP) by means a non-contact tonometer (Topcon CT-1P), slit-lamp biomicroscopy of the anterior segment and dilated fundus examination. All patients were imaged with XR Avanti AngioVue OCTA (Optovue Inc., Fremont, CA). An independent ophthalmologist (GB) was responsible for selecting and exporting high-quality OCT images in accordance with the recommended extension to the OSCAR IB and APOSTEL guidelines for OCT images. Subsequently, the following data were analyzed.

#### ETPKU patients

This study enrolled patients who had received a diagnosis of PKU through neonatal screening. The inclusion criteria for participants were as follows: (i) a verified PKU diagnosis from birth, (ii) age 18 years or older, (iii) of Caucasian descent, (iv) ongoing treatment exclusively through dietary protein restriction, (v) a record of consistent compliance, including regular annual check-ups at the adult metabolic center, and (vi) no previous history of other systemic or ocular disorders. It’s worth noting that, in alignment with European guidelines, all enrolled PKU patients were categorized as having “mild PKU” and demonstrated good adherence to dietary (13, 14) restrictions.

The exclusion criteria were (i) ocular comorbidities such as refractive error over ±5.5 diopters of spherical equivalent, previous ocular trauma, history of ocular disease, e.g., macular degeneration and glaucoma, (ii) history of a systemic condition affecting the retina, e.g., diabetes, hypertension, (iii) any other neurological condition (iv) prematurity (<36 weeks) (v), pregnancy (vi) or other medical treatment potentially interfering with HPA.

#### Case-matched controls

The control group was carefully matched to the ETPKU patient group in terms of age, ethnicity, and gender, as these are well-known factors that can influence OCT and OCTA results. Regarding the matching by age, we considered an interval of birth of 1–2 years. The inclusion criteria for the control group were as follows: (a) of Caucasian ethnicity, (b) no previous history of systemic or ocular disorders, and (c) age 18 years or older. As for the ETPKU patient group, all case-matched controls underwent a comprehensive ophthalmological examination, including BCVA, two consecutive measurements of IOP by means a non-contact tonometer (Topcon CT-1P), slit-lamp biomicroscopy of the anterior segment and dilated fundus examination.

### Outcome measures

The main outcome measures were: (i) RPC density [vessels density (VD) %], and (ii) pRNFL thickness. Secondary outcome measures were: (i) BCVA, (ii) IOP. All patients were imaged with an ultrahigh-resolution spectral domain OCT (SD-OCT) system, specifically the AngioVueTM (RTVue-XR Avanti; Optovue Inc., Fremont, CA, USA, software version 2018.0.0.18). The software automatically segmented the scans, and a single, well-trained examiner performed the measurements on the same day. Intraocular pressures were checked by means a non-contact tonometer (Topcon CT-1P).

### Imaging analysis

OCTA imaging was conducted to assess the radial peripapillary capillary (RPC) plexus across the entire image and peripapillary vessel densities in all study participants. The SD-OCTA device employed in this study had an A-scan rate of 70,000 scans per second, utilized a light source centered at 840 nm, and had a full-width at half maximum bandwidth of 45 nm. To minimize motion artifacts, the RTVue-XR Avanti system acquired two consecutive OCTA volume scans (one horizontal and one vertical), each comprising 400 A-scans, which were then combined. Images from five patients captured using OCTA technology did not meet the required quality standards; therefore, these patients were excluded from the analysis. Additionally, to uphold the high quality of the OCTA images, we applied and adhered to the OSCAR-MP criteria (15).

**Figure 1:**
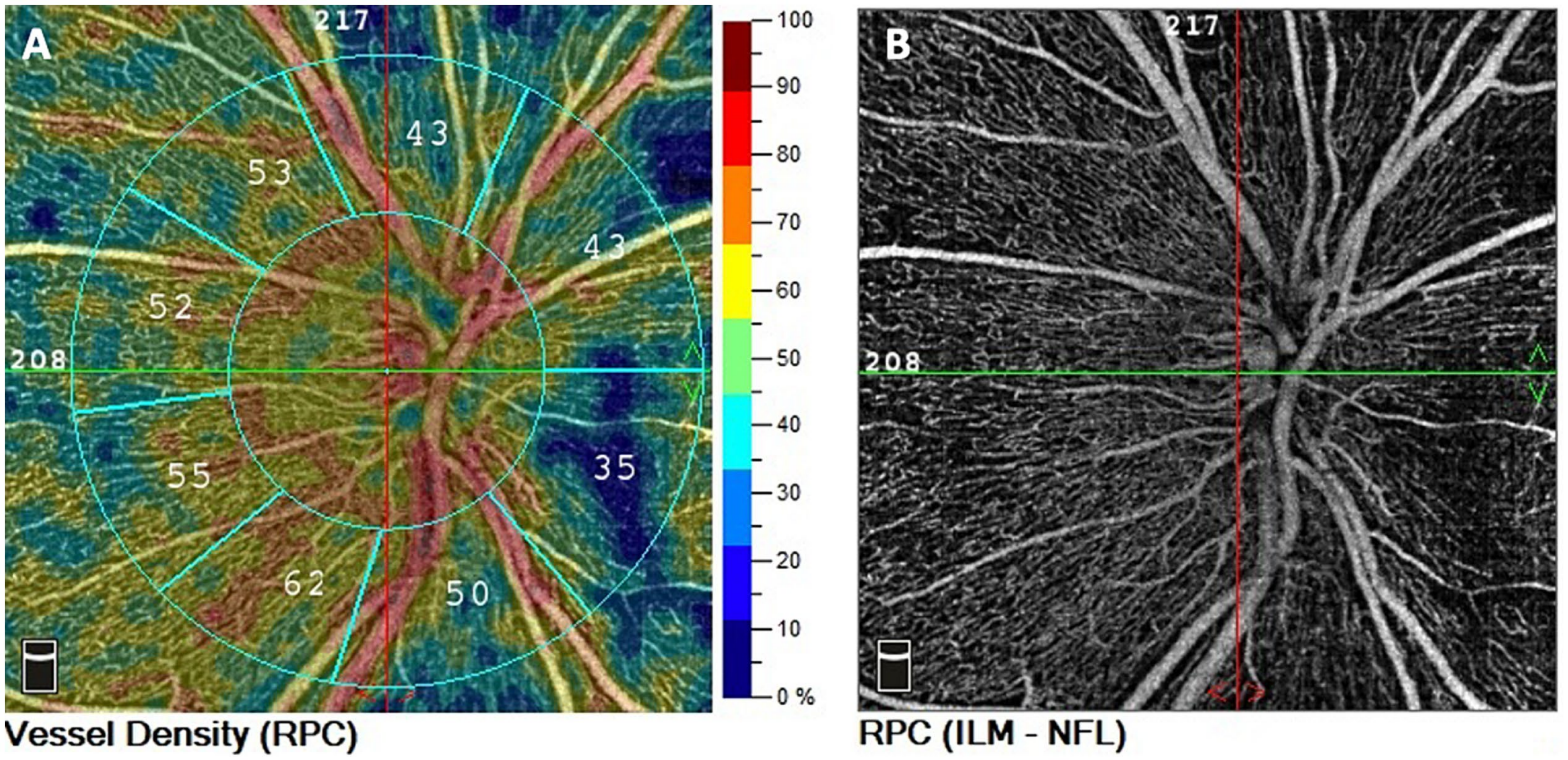
Representation of peripapillary OCT angiography in ETPKU patient. OCTA scan 4.5 × 4.5 mm centered on ONH. **(A)** Color-coded density maps and automatized vessel density measurements of the RPC. **(B)** RPC vascular density image.

A three-dimensional optic disc scan covering an area of 4.5 × 4.5 mm^2, centered on the optic disc, was captured. Vessel density was quantified as the percentage of the area occupied by vessels. Overall and small vessel density were analyzed (Figure 1). The term “small vessel density” pertains to the assessment of the finest peripapillary vessels, excluding the larger vessels, which are instead taken into account in the evaluation of the “overall vessel density.” This analysis is performed automatically by the software within the device.

In cases where segmentation was compromised, manual correction was applied. Additionally, images with poor quality (Signal Strength Index, SSI, less than 6/10), significant motion artifacts, or incorrect segmentation were excluded from the analysis. The pRNFL thickness was obtained using disc map software on the RTvueXR Avanti device, with measurements being repeated until images of at least 9/10 quality were obtained.

### Statistical analysis

All quantitative variables were reported as mean and standard deviation (SD). Data were assessed for normality with Shapiro–Wilk test for all variables. By employing a Sample Size calculator, we determined the power of the sample size to guarantee a 95% confidence level that the true value is within ±5% of the measured/detected value. Differences between the two groups were tested by the independent t test. A Pearson correlation analysis was conducted to examine the correlation between the duration of the disease and the OCT and OCTA parameters. All statistical analyses were performed using Statistical Package for Social Sciences (version 20.0; SPSS Inc., Chicago, IL). The chosen level of statistical significance was *p* < 0.05.

## Results

### Characteristics of patients included in the analysis

36 eyes of 36 patients (18 patients with ETPKU and 18 controls) were included in this analysis. Demographic information and biometric characteristic of the study population are summarized in Table 1. In the patient’s group, the mean age was 30.22 ± 4.02 years (range, 25–36; 10 males,8 females), compared to the control group where the mean age was 29.78 ± 2.48 years (range, 27–36; 10 males,8 females). The eye selected for the study was the one with superior imaging quality. All our patients belonged to the Caucasian ethnicity. We excluded from our sample a ETPKU patient belonging to Asian ethnicity because we could not perform a correct match with a control.

**Table 1:** The clinical and anatomical (OCT) characteristics of subjects included in the analysis.

Variables	Controls (*n* = 18)	ETPKU (*n* = 18)	*p*
Age (years)	30.22 ± 4.02	29.78 ± 2.48	*p* = 0.847
Gender (female, %)	8 (44.44%)	8 (44.44%)	/
Duration of pathology (years)	/	21.3 ± 2.8	/
BCVA	0.01 ± 0.03	0.02 ± 0.04	*p* = 0.278
IOP (mmHg)	14.55 ± 1.8	14.77 ± 2.1	*p* = 0.407
pRNFL (μm)	113.22 ± 13.95	110.78 ± 12.4	*p* = 0.046
RPC, small vessels (VD%)	50.71 ± 3.2	52.31 ± 2.2	*p* = 0.049
Total RPC (VD%)	55.08 ± 3.4	58.5 ± 2.2	*p* < 0.001

Data are presented as Mean ± SD; BCVA, best correct visual acuity; IOP, intraocular pressure; ppRNFL, peripapillary retinal nerve fiber layer; RPC, radial peripapillary capillary.

### Primary outcomes

#### pRNFL thickness

**Figure 2:**
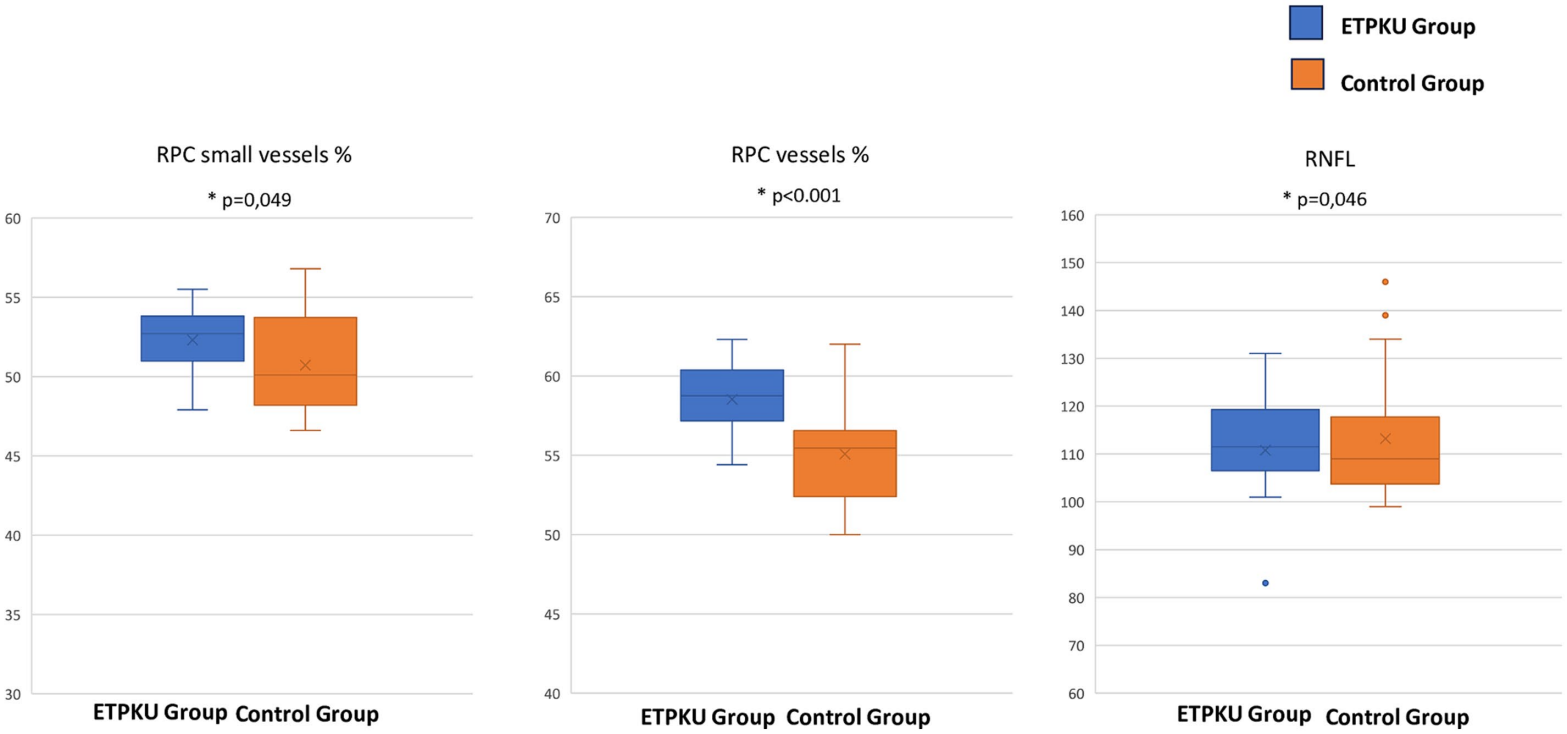
Box and whisker plots showing radial peripapillary capillary plexus vessels density % of the small vessels, of all the plexus and the retinal nerve fiber layer thickness (μm). Each box displays mean (cross within the box), median (central horizontal line) and interquartile range (horizontal extremes of the box) values for each metric. The ends of the whiskers illustrate the minimum and maximum values. Outliers are visualized as dots not included in whiskers. Each graph reports comparisons between the groups for a specific metric (*p* = 0.049, *p* < 0.001, and *p* = 0.046, respectively).

The average pRNFL thickness was 113.22 ± 13.95 μm in controls and 110.78 ± 12.48 μm in patients with ETPKU, significantly reduced compared with controls (*p* = 0.046) (Table 1; Figure 2).

#### RPC vessel density

The mean VD% of the small vessels of the RPC was 52.31 ± 2.2 in ETPKU and 50.71 ± 3.2 in controls (*p* = 0.049), while the VD% of all the RPCP was 58.5 ± 2.2 in ETPKU and 55.08 ± 3.4 in controls (*p* < 0.001) (Table 1; Figure 2).

### Secondary outcomes

#### BCVA and IOP

Mean BCVA was 0.02 ± 0.04 LogMAR in ETPKU and 0.01 ± 0.03 in controls, while mean IOP was 14.77 ± 2.1 in ETPKU and 14.55 ± 1.8 in controls. There were no differences in age, sex, and IOP between the two groups. There was no correlation between main outcomes and duration of the disease (Table 1).

### Correlation between the duration of the pathology and the quantitative OCTA parameters

We investigated the potential association between the duration of the pathology (21.3 ± 2.8 years) and the OCT (pRNFL thickness) and OCTA (RPC density) parameters. Through Pearson correlation analysis, we found no correlation with either pRNFL thickness (*r* = − 0.104, *p* = 0.411) or RPC density (*r* = − 0.089, *p* = 0.248).

## Discussion

In this observational study, we exanimated the potential role of radial peripapillary capillary density and peripapillary retinal nerve fiber layer as markers of retinal damage in individuals with ETPKU compared to age-matched controls. Our findings align with previous research revealing a significant reduction in pRNFL thickness in ETPKU patients. Interestingly, we also observed that ETPKU patients exhibited a higher density of the total RPC compared to controls, suggesting an increased vascular activity associated with the disease.

ETPKU patients, thanks to neonatal screening and the early initiation of life-long phenylalanine-lowering therapy, benefit from protection against the most severe consequences of the disease (16). Nevertheless, ETPKU patients have more pronounced issues in social functioning and neurocognitive problems (17).

Recently, several researchers explored OCT and OCTA findings in ETPKU patients. For instance, Serfozo and coworkers observed significant thinning of both pRNFL and GCC in ETPKU compared to controls. This suggests that ocular microstructural abnormalities in PKU patients might be influenced by dietary control (13). Subsequently, Lotz-Havla and coworkers corroborated these findings. They also noted a reduction of the inner plexiform layer (IPL) volume, and an inner nuclear layer (INL) swelling strongly correlated with the metabolic control (18). These findings are partly consistent with our results, as we did not measure macular thickness, but we observed a thinning in the pRNFL thickness ETPKU patients who were solely treated with dietary control. Several studies have reported a significant correlation between pRNFL thinning and cerebral neurodegeneration, suggesting that average pRNFL might serve as a suitable biomarker for detecting and monitoring the progression of conditions such as Parkinson’s disease (PD) (19). The pattern of impairment observed in PKU, including ETPKU patients, shares similarities with other conditions characterized by dopamine imbalances and white matter damage such as PD or multiple sclerosis (MS) (20). In a recent review from Lancet, the authors stated that measurements of pRNFL and macular GCL and IPL combined could be an important tool for diagnosis, monitoring and research of MS (20). Specifically, in PKU and ETPKU patients, elevated phenylalanine (Phe) levels in the brain can inhibit the activities of enzymes like tyrosine and tryptophan hydroxylases activities, causing a reduction of dopamine (DA) and serotonin biosynthesis (21).

In the eye, DA is released by amacrine cells, activates dopamine receptors distributed throughout the retina, and covers multiple trophic roles related to circadian rhythmicity, cell survival, and eye growth (22). Consequently, conditions of metabolic dysregulation of DA like PKU, have been showed to be related to a DA imbalance in the retina as well (23). The exact mechanism of neurotoxicity on the retina in PKU is not clearly understood, but, according to one of the hypotheses, altered DA levels and impaired neurotransmission, are discussed to play a role in the retinal damage (21).

In addition to using OCT angiography, this study also evaluated the perfusion of the peripapillary vascular plexus. A previous OCTA study on ETPKU individuals by Serfozo et al. revealed a decrease in parafoveal superficial capillary plexus density in ETPKU compared to controls (24). However, our findings demonstrated a significant increase in RPC perfusion in ETPKU patients.

## Future direction

Given the aforementioned common pathways of retinal damage among DA imbalance diseases, it is not surprising the increased RPC density we found in ETPKU patients compared to controls (25). Indeed, the RPC is a network of capillaries that has a unique anatomic organization since it runs in parallel with retinal nerve fiber layer axon bundles and plays a role in nourishing the axons of retinal ganglion cells in peripapillary area (24).

We speculate that the neuroinflammation and the cellular stress may lead to an increased amount of vasodilatory and vasogenic mediators, resulting in enhanced blood flow to pRNFL axons. A similar mechanism has been proposed in Parkinson’s disease (26). This hypothetical condition might explain the simultaneous increase in RPC density and pRNFL thinning we found. Another potential explanation may be the formation of dysfunctional capillaries similar to the string vessels of Alzheimer’s disease.

The present study has limitations to consider when interpreting our findings. Our sample size was relatively small, which may limit the results generalizability. Furthermore, it’s important to acknowledge that our analysis focused solely on one phenotype of these patients (ETPKU), which may introduce a potential bias in our results. We analyzed OCTA images using spectral domain OCTA which utilizes shorter wavelength light in comparison with swept source OCT angiography. We did not measure the macular thickness and we did not compare the retinal measurements with brain parameters correlated to neurocognitive impairment. Additionally, we did not investigate the correlation between the duration of the pathology and the quantitative OCTA parameters analyzed.

Future randomized prospective case–control trials are necessary to confirm our findings, as well as longitudinal studies are always recommended for establishing the validity of such biomarkers.

In conclusion, our observational study provides evidence of neurovascular impairment in ETPKU patients who were solely treated with dietary control when compared to controls. Through structural OCT and OCT angiography, we observed thinning of the peripapillary nerve fibers accompanied by an increase in perfusion of the peripapillary vascular plexus. We suggest that, similar to other neurodegenerative diseases, increased neuroinflammation may lead to a higher production of vasodilatory mediators, resulting in an augmented vessel density in the RPC. These findings warrant further investigation as they may serve as an additional retinal biomarker in individuals with ETPKU.

## Data availability statement

The original contributions presented in the study are included in the article/supplementary material, further inquiries can be directed to the corresponding author.

## Ethics statement

Ethical review and approval was not required for the study on human participants in accordance with the local legislation and institutional requirements. Written informed consent to participate in this study was provided by the participants.

## Author contributions

RB: Writing – original draft. GB: Formal analysis, Methodology, Supervision, Writing – review & editing. MGa: Data curation, Writing – original draft. SG: Supervision, Writing – original draft. RF: Data curation, Writing – original draft. AM: Conceptualization, Writing – original draft. MGr: Conceptualization, Writing – review & editing. MP: Data curation, Writing – original draft. PP: Conceptualization, Writing – original draft. EG: Conceptualization, Writing – review & editing. MD’a: Data curation Writing – original draft. GA: Conceptualization, Writing – review & editing. FB: Conceptualization, Writing – review & editing. PV: Supervision, Writing – review & editing.
